# The Use of Biomarkers and Genetic Screening to Diagnose Frontotemporal Dementia: Evidence and Clinical Implications

**DOI:** 10.3389/fnins.2019.00757

**Published:** 2019-08-06

**Authors:** Helena Gossye, Christine Van Broeckhoven, Sebastiaan Engelborghs

**Affiliations:** ^1^Neurodegenerative Brain Diseases Group, Center for Molecular Neurology, VIB, Antwerp, Belgium; ^2^Institute Born – Bunge, University of Antwerp, Antwerp, Belgium; ^3^Department of Neurology and Center for Neurosciences, UZ Brussel and Vrije Universiteit Brussel, Brussels, Belgium

**Keywords:** dementia, frontotemporal dementia, Alzheimer, biomarker, genetics, cerebrospinal fluid, MRI

## Abstract

Within the wide range of neurodegenerative brain diseases, the differential diagnosis of frontotemporal dementia (FTD) frequently poses a challenge. Often, signs and symptoms are not characteristic of the disease and may instead reflect atypical presentations. Consequently, the use of disease biomarkers is of importance to correctly identify the patients. Here, we describe how neuropsychological characteristics, neuroimaging and neurochemical biomarkers and screening for causal gene mutations can be used to differentiate FTD from other neurodegenerative diseases as well as to distinguish between FTD subtypes. Summarizing current evidence, we propose a stepwise approach in the diagnostic evaluation. Clinical consensus criteria that take into account a full neuropsychological examination have relatively good accuracy (sensitivity [se] 75–95%, specificity [sp] 82–95%) to diagnose FTD, although misdiagnosis (mostly AD) is common. Structural brain MRI (se 70–94%, sp 89–99%) and FDG PET (se 47–90%, sp 68–98%) or SPECT (se 36–100%, sp 41–100%) brain scans greatly increase diagnostic accuracy, showing greater involvement of frontal and anterior temporal lobes, with sparing of hippocampi and medial temporal lobes. If these results are inconclusive, we suggest detecting amyloid and tau cerebrospinal fluid (CSF) biomarkers that can indicate the presence of AD with good accuracy (se 74–100%, sp 82–97%). The use of P-tau_181_ and the Aβ_1__–__42_/Aβ_1__–__40_ ratio significantly increases the accuracy of correctly identifying FTD vs. AD. Alternatively, an amyloid brain PET scan can be performed to differentiate FTD from AD. When autosomal dominant inheritance is suspected, or in early onset dementia, mutation screening of causal genes is indicated and may also be offered to at-risk family members. We have summarized genotype–phenotype correlations for several genes that are known to cause familial frontotemporal lobar degeneration, which is the neuropathological substrate of FTD. The genes most commonly associated with this disease (*C9orf72, MAPT, GRN, TBK1*) are discussed, as well as some less frequent ones (*CHMP2B, VCP*). Several other techniques, such as diffusion tensor imaging, tau PET imaging and measuring serum neurofilament levels, show promise for future implementation as diagnostic biomarkers.

## Introduction

Frontotemporal lobar degeneration (FTLD) represents a group of neurodegenerative brain diseases, characterized by relatively localized degeneration of the frontal and anterior temporal lobes. One of the clinical entities associated with FTLD is frontotemporal dementia (FTD), a neurodegenerative brain disorder with a diverse clinical presentation and multiple possible molecular pathways of disease. The prevalence of FTD is 1 to 461 per 100.000 individuals, accounting for approximately 2.7% of all dementias (Coyle-Gilchrist et al., 2016; Hogan et al., 2016). In patients under 65 years, FTD accounts for approximately 10.2% of all dementias, and is the second most common dementia subtype after Alzheimer’s disease (AD) in this age group (Hogan et al., 2016).

Clinically, patients with FTLD display a progressive change in behavior, so-called behavioral variant FTD (bvFTD), and/or decline of language, or language variant presenting as primary progressive aphasias (PPA), such as semantic variant PPA (sv-PPA), non-fluent PPA (nfv-PPA), and logopenic PPA (lv-PPA) (Rascovsky et al., 2011). There may be a symptomatic overlap with atypical parkinsonian disorders or motor neuron disease (MND) (Chare et al., 2014).

Upon post-mortem examination of the affected brain, FTLD is characterized by protein inclusions in degenerating neurons. The composition of these inclusions varies across the disease spectrum. The majority of patients (85%) show cellular inclusion bodies that are comprised of either tau (FTLD-tau) or trans-active response DNA binding protein of 43 kDa (TDP-43) (FTLD-TDP). The latter can be subdivided into FTLD-TDP A to E (Mackenzie et al., 2010; Van Mossevelde et al., 2018). Another subgroup of cases present with inclusions of the fused in sarcoma (FUS) protein (FTLD-FUS). In the remaining cases, inclusions are comprised of (hitherto unidentified) proteins of the ubiquitin proteasome system (FTLD-UPS) or, infrequently, no protein inclusions are found (FTLD-ni) (Mackenzie et al., 2010; Sieben et al., 2012). The latter has also been described as dementia lacking distinct histopathology (DLDH), a term introduced by Knopman et al. (1990) in patients with degeneration of the brain without the presence of neuronal inclusions or senile plaques. Many of these cases have since been reclassified, as they were later found to have neuronal inclusions staining positive for ubiquitin (Mackenzie et al., 2006). The underlying disease mechanisms involved in this rare subtype are not yet fully understood.

In FTD, heritability plays a main role, with a positive family history in 39–50% of cases (Rosso et al., 2003; Rohrer et al., 2009; Sieben et al., 2012; Po et al., 2014). An autosomal dominant presentation is seen in 10–23% of patients (Goldman et al., 2005; Rohrer et al., 2009; Sieben et al., 2012). The most frequent mutated genes involved in FTD with a dominant inheritance pattern are the *C9orf72* (8.2%) (DeJesus-Hernandez et al., 2011; Renton et al., 2011; Gijselinck et al., 2012), the progranulin (*GRN*) (4.1%) (Baker et al., 2006; Cruts et al., 2006; Sieben et al., 2012) and the microtubule associated protein tau (*MAPT*) (5.6%) (Hutton et al., 1998). Less frequent disease genes are those coding for the protein fused in sarcoma (*FUS*) (Yan et al., 2010; Deng et al., 2014), chromatin-modifying protein 2b (*CHMP2B*) (van der Zee et al., 2008; Isaacs et al., 2011), TAR DNA-binding protein (*TARDBP*) (Lattante et al., 2013), TANK binding kinase 1 (*TBK1*) (Cirulli et al., 2015; Freischmidt et al., 2015; Gijselinck et al., 2015; Pottier et al., 2015), valosin containing protein (*VCP*) (Watts et al., 2004), sequestosome 1 (*SQSTM1*) (Fecto et al., 2011), and several others (Po et al., 2014; Woollacott and Rohrer, 2016; Che et al., 2018).

Amongst all of these heterogeneous subtypes in multiple domains, significant correlations can be found between causal gene, neuropathology and a certain set of clinical presentations. However, a one-to-one relationship is lacking (Sieben et al., 2012; Van Mossevelde et al., 2018).

The diagnosis of bvFTD and of the different language variants of FTD is most commonly based upon clinical diagnostic criteria (Gorno-Tempini et al., 2011; Rascovsky et al., 2011). These are based on the presenting core symptoms, complemented with results of (a combination of) brain magnetic resonance imaging (MRI), 18-fluorodeoxyglucose (FDG) positron emission tomography (PET) scan, perfusion single-photon emission tomography (SPECT) scan and DNA screening for causal mutations.

Several other techniques can aid in the differential diagnosis of FTD, especially when the clinical presentation is suggestive for other types of dementia as illustrated by our clinical vignette. To distinguish FTD from dementia caused by AD, cerebrospinal fluid (CSF) biomarkers demonstrating amyloid and tau pathology and amyloid tracer imaging techniques are widely used in clinical practice (Croisile et al., 2012; Dubois et al., 2014). Although these CSF biomarkers have a good diagnostic accuracy for AD, some cases display an atypical biochemical signature, as we will discuss below. Numerous other novel techniques are hitherto primarily carried out in a research setting.

Due to its heterogeneous nature, the diagnosis of FTD can be challenging. This review aims to summarize the state of the art in the current wide range of available diagnostic tools, covering neuropsychological evaluation, neurochemical and imaging biomarkers and genetic testing. More specifically, we will summarize the indications, evidence and added diagnostic and therapeutic value of these techniques within the framework of a clinical setting.

**Clinical Vignette**

A 65-year old patient consulted the neurologist with complaints of gradually progressive memory deficits.

Her previous medical history included arterial hypertension, surgical removal of an occipital meningioma and melanoma. Her medication intake was limited to a diuretic and a beta-blocker.

The presenting complaints were those of short term memory problems with insidious onset, progressively worsening over the course of 1 year. There was also occasional occurrence of diminished orientation in space.

The family history revealed that the maternal grandfather had late-onset dementia.

Clinical neurological examination was perfectly normal except for the presence of palmomental reflexes. No other frontal release signs, parkinsonism or any other pathological sign was found.

**FIGURE 1:**
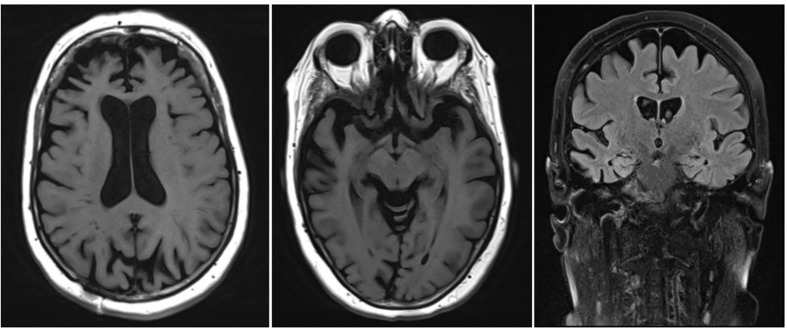
Brain MRI of the patient described in the clinical vignette.

A diagnostic workup was performed:

• An extensive blood analysis showed no anomalies except for a slight macrocytic anemia. Thyroid function and blood vitamin levels were adequate.
• Neuropsychological testing revealed a single domain amnestic mild cognitive impairment.
• An MRI scan of the brain showed corticosubcortical atrophy, more than what would be expected for the patient’s age (Figure 1).

Based upon initial anamnestic presentation, the suspicion for prodromal early onset AD was raised.

During the next encounter, the patient was accompanied by her husband who mentioned that 1 year prior to the onset of the memory complaints, he had begun to notice behavioral changes. There was slight disinhibition, with the tendency to laugh at socially inappropriate occasions. In addition, the patient had developed apathy, of which loss of initiative was the most prominent symptom. There were some dysexecutive symptoms, with difficulties managing her everyday tasks. These symptoms had implications on the course of the activities of daily living (ADL), with a lack of personal hygiene and self-care. Only later during the disease course did the memory complaints also become apparent.

**FIGURE 2:**
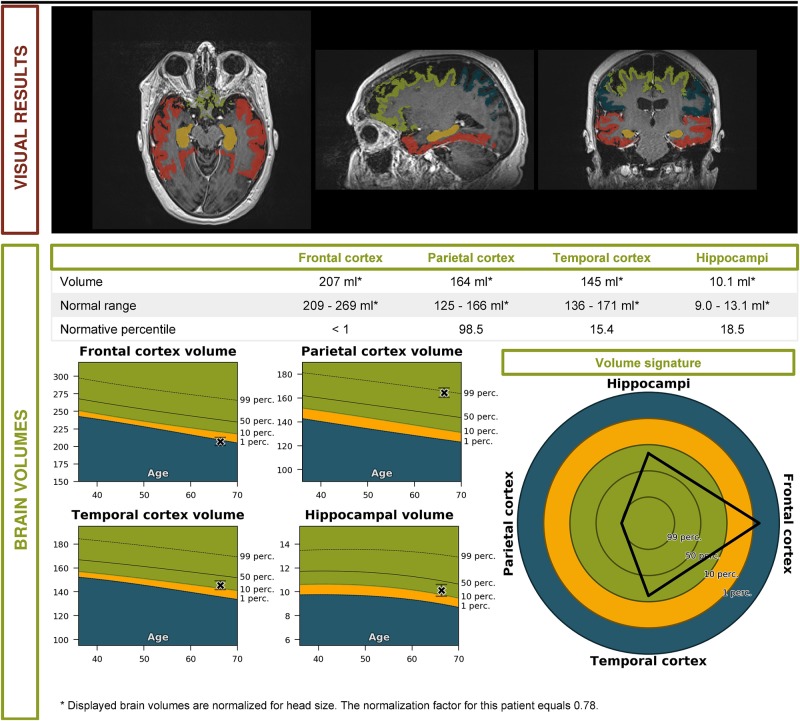
Volumetric brain MRI of the patient described in the clinical vignette (report by Icometrix).

Paraclinical re-evaluation of the patient was performed:

• An automated volumetric analysis of the brain MRI scan showed a more pronounced atrophy of the frontal lobes in comparison with age-matched controls. The hippocampal volume was normal for age (Figure 2).
• On an FDG PET scan of the brain, a significantly lower metabolism could be visualized anteromedially in the right frontal lobe and in the right temporal lobe. Minimal hypometabolism was found on the left side as well.
• CSF biomarker analysis revealed elevated T-tau (632 pg/ml; normal: <501 pg/ml) and P-tau (69.5 pg/ml; normal: <57 pg/ml) levels with normal values of Aβ_1__–__42_ (1551 pg/ml; normal: >755 pg/ml) and Aβ_1__–__42_/Aβ_1__–__40_ ratio (0.196; normal: >0.106). This result indicated the presence of a neurodegenerative brain disease, but made the diagnosis of an underlying AD less likely.
• Genetic testing of known causal AD and FTD genes was performed and could not identify a causal mutation.

These biomarkers were incompatible with the diagnosis of prodromal AD. Furthermore, a striking localized atrophy in the frontal lobe on the automated volumetric analysis of the brain MRI scan (Figure 2) and a significantly lower metabolism in the right frontal and temporal lobes prompted the shift of focus to a possible FTD.

Based on the presenting clinical symptoms and the CSF and imaging biomarkers, the tentative diagnosis of behavioral variant FTD (bvFTD) was retained.

This case serves as an example of the importance of an extensive patient history as well as of specific biomarkers for the diagnosis of neurodegenerative brain disorders. Underneath an atypical clinical presentation, highly suggestive for prodromal AD, an underlying FTD could be unveiled.

## Clinical Diagnostic Criteria for FTD: Strengths and Limitations

The most recently revised consensus criteria for the clinical diagnosis of bvFTD are those by Rascovsky et al. (2011). In this hierarchical framework, three levels of diagnostic certainty are distinguished. The first degree determines whether or not the term “possible” bvFTD is appropriate and is based upon presence of core symptoms (behavioral disinhibition, apathy/inertia, compulsive behavior, dietary changes and executive dysfunction with spared memory and visuospatial functions) alone. For “probable” and “definite” bvFTD, results of imaging and histopathology/DNA analysis, respectively, are taken into account. The consensus criteria for possible bvFTD have a sensitivity of 85–95% and a specificity of 82%, irrespective of the underlying proteinopathy (Rascovsky et al., 2011; Harris et al., 2013; Balasa et al., 2015). For probable bvFTD, sensitivity and specificity values are 75–85 and 95%, respectively (Rascovsky et al., 2011; Harris et al., 2013; Balasa et al., 2015). A higher sensitivity is reached in early onset dementia compared with late onset, as in younger age groups there is significantly more disinhibition, loss of empathy and compulsive behavior (Rascovsky et al., 2011; Balasa et al., 2015). False positive diagnoses are most common in patients with a later onset, an absence of family history for dementia and a more apathetic presentation. They mainly turn out to be AD upon neuropathological examination (Harris et al., 2013; Balasa et al., 2015).

Several other clinical tools exist to measure frontal lobe dysfunction and therefore differentiate between FTD and AD. Many assessment batteries of neurobehavioral symptoms, such as the Schedules for Clinical Assessment in Neuropsychiatry (Wing et al., 1990), the Scale for Emotional Blunting (Mendez et al., 2006), the Middelheim Frontality Score (De Deyn et al., 2005) and the Frontal Behavior Inventory (Kertesz et al., 2000) have a good discriminative ability (Milan et al., 2008; Mathias and Morphett, 2010).

Similar consensus criteria, with a stepwise approach to the level of evidence, exist for the language variants of FTD (Gorno-Tempini et al., 2011). The initial and most prominent symptoms should be deficits in language for the diagnosis of PPA to be considered. Subsequently, aphasia is characterized more specifically, distinguishing three separate entities: sv-PPA presenting with impaired comprehension but spared speech production, nfv-PPA with agrammatisms and speech apraxia, and lv-PPA with anomia and impaired single-word comprehension. The latter is only very rarely associated with FTLD; more commonly (77%) is it seen in patients with an underlying AD pathology (Chare et al., 2014; Leyton et al., 2015; Spinelli et al., 2017).

Both false-positive and false-negative diagnoses of FTD are most often confounded with AD (Harris et al., 2013). Analysis of a large neuropathological confirmed cohort brought to light that several clinical characteristics can discriminate FTD from AD with great accuracy (>86%); these were word finding difficulties, phonological errors, delusions and lack of object agnosia for AD, and relative lack of neuropsychiatric features, phonological errors and gait disturbance for FTD. Even then, about 36% of AD cases could not be differentiated from FTD based on clinical diagnostic criteria (Chare et al., 2014). 52% of AD patients with an atypical profile (commonly called “behavioral variant AD”) meet the criteria for possible FTD, with apathy as the main overlapping feature (Ossenkoppele et al., 2015). The notion that deficits in episodic memory can reliably distinguish FTD from AD used to be considered a strong criterion (Neary et al., 1998). However, many FTD patients do initially present with complaints of memory loss and often meet AD consensus criteria (Khachaturian, 2011; Fernandez-Matarrubia et al., 2017). Apart from AD, there is also substantial symptomatic overlap between FTD and MND, with co-occurrence of MND in 12.5–15% of FTD patients (Burrell et al., 2011; Saxon et al., 2017).

In many cases, some degree of parkinsonism is present upon initial presentation. When language and behavioral symptoms overlap, there is often a difficult differential diagnosis between so-called FTD with parkinsonism syndromes and atypical parkinsonian syndromes such as corticobasal degeneration (CBD) and progressive supranuclear palsy (PSP) (Espay and Litvan, 2011).

Another gray area exists along the borders of psychiatric disorders. An important limitation of the clinical diagnostic criteria is their relatively subjective and arbitrary nature. Symptoms like aberrant behavior are prone to observer bias, and are usually described by informants, making them inherently subjective and creating another layer of interaction in which nuances may be lost. Apathy, emotional withdrawal, hallucinations, delusions, psychosis, and compulsive behaviors can also easily be misdiagnosed as part of a psychiatric disorder such as depression, schizophrenia or bipolar disorder, especially with early onset dementia (Waldo et al., 2015).

In summary, these commonly used sets of clinical criteria offer a framework for the standardized classification of patients with FTD into subtypes. However, overlap between categories and with other disorders may exist as well as mixed presentations, as the disease progresses and new symptoms arise.

## Imaging Techniques to Diagnose FTD

### Structural Brain MRI

A typical first step in obtaining an imaging-supported diagnosis of FTD is structural brain MRI. T1-weighted MRI images aid in evaluating (localized) brain atrophy, while flair images provide proof of possible vascular damage. In FTD, one typically expects a more pronounced loss of volume in the frontal lobes and the anterior temporal lobes (Varma et al., 2002; Boccardi et al., 2003; Kipps et al., 2007; Meyer et al., 2017; Che et al., 2018). Asymmetry is not uncommon, usually with a greater involvement of the dominant hemisphere, especially in language variants (Boccardi et al., 2003; Kipps et al., 2007; Che et al., 2018). The hippocampi and medial temporal lobes are typically relatively spared (Varma et al., 2002; Boccardi et al., 2003; Kipps et al., 2007; Meyer et al., 2017; Che et al., 2018).

The aforementioned clinical subtypes of FTD generally have a distinct MRI atrophy pattern, although overlap exists. bvFTD patients typically show atrophy in the dorsolateral, orbital and medial frontal cortices as well as other regions of the salience network (SN) (Che et al., 2018). sv-PPA patients show (often subtle) unilateral atrophy of the anterior temporal pole, with a characteristic “knife-edge” aspect in this area. Left-sided atrophy usually comes with a typical sv-PPA symptomatology, while patients with a predominantly right-sided atrophy tend to mimic bvFTD (Rabinovici and Miller, 2010). The former is most common, but involvement of the contralateral side tends to occur within 3 years (Rabinovici and Miller, 2010). nfv-PPA patients typically show volume loss in the left perisylvian region, more specifically the frontal operculum, premotor, and supplementary motor areas as well as the insula. Involvement of Broca’s area explains the motor component of the aphasia (Pressman and Miller, 2014; Che et al., 2018). Comparable relatively specific patterns of atrophy exist within groups of carriers of the same causal mutation for FTD (Che et al., 2018; Van Mossevelde et al., 2018).

Sensitivity (se) and specificity (sp) of brain MRI for differentiating FTD from controls have been reported as 70–94 and 89–99%, respectively (Likeman et al., 2005; Harper et al., 2016; Vijverberg et al., 2016). Structural brain MRI is especially useful in distinguishing FTD from AD, with a sensitivity of 55–94% and a specificity of 81–97% by comparing patterns of atrophy (Boccardi et al., 2003; Likeman et al., 2005; Harper et al., 2016). FTD patients show significantly more frontal and (mostly anterior) temporal atrophy with relative sparing of the hippocampus, while AD patients typically show bilateral hippocampal and medial temporal lobe atrophy, with relative sparing of the frontal lobes. Asymmetrical involvement of the hemispheres is another feature that is common in FTD patients and rare in AD (Varma et al., 2002; Boccardi et al., 2003; Likeman et al., 2005). The left frontal and temporal regions are more often involved than the right in FTD (Boccardi et al., 2003). One study found that asymmetrical involvement of the brain differentiated FTD from non-FTD with a sensitivity of 38% and a specificity of 100% (Varma et al., 2002).

In the early stages of the disease, however, a brain MRI scan may not yet show abnormalities (Meeter et al., 2017). One study showed an incongruence between clinical subtypes: almost half of bvFTD patients had normal MRI scans, while all of the sv-PPA patients displayed a pathological scan. However, not all scans analyzed were made at initial presentation (Kipps et al., 2007). The presence of MRI abnormalities seems to be associated with more severe imitation behavior and disinhibition compared to those patients with normal MRI (Koedam et al., 2010). On the other hand, a large-scale trial examining presymptomatic carriers of a known causal mutation was able to find structural imaging changes 5–10 years before expected onset, based on the average onset within the family. The insular and temporal cortices were the earliest regions to show atrophy (Rohrer et al., 2015).

Comparing brain atrophy and abnormalities is classically done by visual evaluation with commonly used scales such as the rating scale for medial temporal lobe atrophy (MTA) (Scheltens et al., 1995), the global cortical atrophy (GCA) scale (Pasquier et al., 1996) and the Fazekas’ scale (Fazekas et al., 1987). However, novel fully automated image quantification methods such as automated volumetry, voxel-based morphometry (VBM), tensor-based morphometry (TBM), manifold learning, region of interest (ROI)-based grading and automated measuring of vascular burden are increasingly used. One study (Koikkalainen et al., 2016) showed an accuracy rate of respectively 50, 65, 64, 50, 58, and 33% for these techniques in differentiating between normal controls, AD, FTD and Lewy body dementia (LBD). However, in combining different techniques, accuracy rates of 69% were reached. FTD patients were most often misclassified as AD patients (21% of cases). Here, visual MRI ratings reached an accuracy of 52%, scoring significantly worse than combined automated quantification methods (Koikkalainen et al., 2016). A 2017 multi-centric MRI study found an accuracy rate of 85% for the diagnosis of bvFTD using automatic pattern recognition algorithms (Meyer et al., 2017).

A study by Munoz-Ruiz et al. (2012) compared hippocampal volumetry (HV) with VBM and TBM in differentiation between controls, FTD patients and AD patients. FTD patients were rather accurately distinguished from controls with all techniques (se and sp were 84 and 80% for HV, 80 and 71% for TBM, 87 and 81% with VBM). VBM was most suitable for the differential diagnosis of FTD versus AD, with a sensitivity of 72% and a specificity of 67% (Munoz-Ruiz et al., 2012). HV alone might be suitable to distinguish between FTD or AD and healthy controls, but in itself is not sufficient to differentiate between the two types of dementia (se and sp both 55%); here, a more extensive screening of the pattern of atrophy is needed (Munoz-Ruiz et al., 2012; de Souza et al., 2013).

These novel MRI techniques may aid in correctly identifying atypical presentations of neurodegenerative brain diseases. In AD patients with a predominantly behavioral or dysexecutive presentation, VBM shows marked atrophy in bilateral temporoparietal regions and only limited atrophy in the frontal cortex compared to controls, a pattern strikingly similar to AD patients with a typical presentation (Ossenkoppele et al., 2015).

Another relatively new tool is the automated measurement of cortical thickness, which shows a specific pattern of cortical thinning in FTD compared to AD. This technique has similar results in differentiating FTD from AD as the more classically measured cortical volume (Du et al., 2007). It is also useful to distinguish between clinical subtypes of FTD. One study showed distinct differences in cortical thinning between nfv-PPA and sv-PPA, resulting in an accurate diagnosis of 90% of cases (Agosta et al., 2015).

### Brain FDG PET and Perfusion SPECT Scan to Diagnose FTD

Another part of the investigation of patients with suspected FTD is based on functional imaging of the brain (Rascovsky et al., 2011). A commonly used technique is the FDG PET scan, which visualizes the cerebral glucose metabolism. Another method for functional imaging is perfusion SPECT, which also requires the intravenous injection of a radiolabeled tracer molecule, but instead is a measure of cerebral blood flow (CBF). The radiotracers most commonly used are ^99m^technetium-hexamethylpropylenamine oxime (^99m^Tc-HMPAO) or ^99m^technetium-ethyl-cysteinate dimer (^99m^Tc-ECD) (Archer et al., 2015).

In FTD, hypometabolism and hypoperfusion are typically seen in the frontal and anterior temporal lobes, more specifically in bilateral medial, inferior and superior lateral frontal cortices, anterior cingulate, left temporal, and right parietal cortices and the caudate nuclei. Usually, the hypometabolism correlates with, but often precedes, the atrophy on MRI (Varma et al., 2002; Le Ber et al., 2006; Morbelli et al., 2016). Local hypometabolism is best observed in the frontal regions for bvFTD, temporal regions for sv-PPA and perisylvian regions for nfv-PPA (Che et al., 2018).

For FTD, the sensitivity of FDG PET scan ranges from 47 to 90%; the specificity from 68 to 98% (Foster et al., 2007; Kerklaan et al., 2014; Vijverberg et al., 2016). In subjects with late onset behavioral changes, bvFTD could be differentiated from other diagnoses with a sensitivity of 96% and a specificity of 73% when combined with structural MRI (Vijverberg et al., 2016). An increase of the abnormalities can be seen over time, indicating the potential usefulness of FDG PET as a biomarker of disease progression (Diehl-Schmid et al., 2007).

The sensitivity and specificity of SPECT for the differential diagnosis between FTD and AD were reported as 36–100 and 41–100%, respectively (Dougall et al., 2004; Yeo et al., 2013; Archer et al., 2015). A Belgian study comparing SPECT abnormalities between cohorts of FTD and AD patients showed that biparietal hypoperfusion was significantly more present in AD, while bifrontal hypometabolism was significantly associated with FTD. 74% of AD patients and 81% of FTD patients were correctly classified (Pickut et al., 1997). Another study showed that severely decreased frontal (se 76%, sp 60%) and temporal (se 71%, sp 55%) CBF and asymmetry between hemispheres (se 38%, sp 73%) were good markers to differentiate FTD from AD and vascular dementia (VaD) (Varma et al., 2002).

Thus, brain FDG PET or SPECT scans can significantly increase diagnostic accuracy and have the advantage of showing abnormalities fairly early in the disease process (Varma et al., 2002; Morbelli et al., 2016). A systematic review summarizing the evidence on the comparison of PET and SPECT for the diagnosis of neurodegenerative brain diseases showed that some studies find the techniques equally useful, while others describe better results with PET. However, there is a lack of methodologically good direct comparative studies. SPECT has the advantage of being more affordable whereas PET has a better spatial resolution (Davison and O’Brien, 2014).

## Neurochemical Biomarkers to Diagnose FTD

### Core AD CSF Biomarkers: Aβ_1__–__42_, Aβ_1__–__42_/Aβ_1__–__40_ Ratio, T-tau and P-tau

The currently most used panel to assess pathological markers of neurodegenerative brain disease is a combination of amyloid-β of 42 amino acids (Aβ_1__–__42_), total tau protein (T-tau), and hyperphosphorylated tau (P-tau_181_) in CSF (Engelborghs et al., 2008; Bjerke and Engelborghs, 2018; Niemantsverdriet et al., 2018). They are not, in fact, used for the diagnosis of FTD, but rather to make the diagnosis of AD less likely when doubt exists. A CSF biomarker profile characteristic for AD, with good diagnostic accuracy (>80%), shows decreased Aβ_1__–__42_ values, in combination with increased T-tau and P-tau values (Engelborghs et al., 2008; Bjerke and Engelborghs, 2018). Some patients do not completely match this typical AD CSF profile. As amyloid pathology is measurable much earlier in the disease process than tau pathology, there may be an isolated decrease in Aβ_1__–__42_ in early disease stages (Blennow and Zetterberg, 2018). CSF tau markers are more strongly associated with cognitive decline and disease progression in AD than Aβ_1__–__42_ (Niemantsverdriet et al., 2017b). The opposite may also be true, as between-individual variations in total Aβ production or secretion from neurons and variations in CSF dynamics may cause Aβ_1__–__42_ to fall within a normal range, while underlying amyloid pathology is present (Blennow and Zetterberg, 2018).

In a large (*n* = 78) neuropathologically confirmed Belgian cohort of AD and non-AD dementia patients, the added diagnostic value for AD versus non-AD dementia (FTD amongst others) of the standard CSF biomarker panel to clinical consensus criteria was measured. In patients with an ambiguous (AD versus non-AD dementia) clinical diagnosis, the correct diagnosis would have been established in 67% of cases. As the diagnosis based on clinical consensus criteria was straightforward, no added diagnostic value could be measured. A misdiagnosis based on CSF biomarkers often occurs in patients with non-AD and AD co-pathology (Niemantsverdriet et al., 2017b, 2018). Apart from this, T-tau may also be significantly increased after stroke and in Creutzfeldt–Jakob’s disease; whereas P-tau_181_ is a more specific CSF biomarker for AD. P-tau_181_ is indispensable for the differential diagnosis between AD and non-AD neurodegenerative brain disorders. Both Aβ_1__–__42_ and T-tau may be abnormal at intermediate levels in DLB, FTD, VaD, and CJD (Niemantsverdriet et al., 2017b, 2018). Sensitivity and specificity to distinguish FTD from AD have been reported as 74–100 and 82–97%, respectively (Irwin et al., 2013).

To further improve diagnostic performance for AD, the use of the Aβ_1__–__42_/Aβ_1__–__40_ ratio has been proposed with good results (Janelidze et al., 2016; Leuzy et al., 2016; Niemantsverdriet et al., 2017a; Bjerke and Engelborghs, 2018), improving accuracy with 14–36% compared to Aβ_1__–__42_ alone (Janelidze et al., 2016; Lewczuk et al., 2017). An isolated decrease in Aβ_1__–__42_ is more specific to AD, while a global decrease in both Aβ isoforms may be correlated with subcortical damage in general or may even be due to interindividual variability (Janelidze et al., 2016; Lewczuk et al., 2017; Niemantsverdriet et al., 2017a).

Cerebrospinal fluid biomarker analysis of the aforementioned proteins is fairly cost-effective (Niemantsverdriet et al., 2017b). A disadvantage of all CSF biomarkers is the necessity to perform a lumbar puncture (LP). However, when performed correctly, LP has a low complication rate and a fairly good tolerability (Duits et al., 2016; Engelborghs et al., 2017). Overall, evidence shows a clear indication for the use of CSF biomarkers Aβ_1__–__42_, Aβ_1__–__42_/Aβ_1__–__40_ ratio, T-tau and P-tau_181_ as an efficient measure to confirm or rule out AD when other biomarkers are inconclusive. FTLD cannot be diagnosed based on CSF biomarkers yet; non-specific, intermediate decreased Aβ_1__–__42_ and increased T-tau CSF levels may or may not be present.

## Genetic Screening for Known Causal Genes for FTD

It has been suggested that the presence of a pathogenic mutation in an FTD gene in a patient with suspected FTD should be enough to confirm a diagnosis of “definite” FTD, putting genetic screening at the same level of diagnosis as autopsy brain histopathology analysis (Rascovsky et al., 2011). However, the absence of a mutation does not contribute to the diagnosis since FTD is frequently sporadic and FTD genes do not explain all families with FTD.

To elucidate further the value of genetic screening in clinical practice, we briefly outlined the known genotype–phenotype correlations of pathological mutations in the common FTD genes, including average age at onset and disease duration (overview in Table 1). We also mentioned the neuropathological correlation expected with each mutated gene, which is of particular interest when new disease-modifying therapies become available. The discovery of a mutation might mean a prognosis for the patient carrier and the presymptomatic family members. It may also guide the clinician in coupling a presenting clinical phenotype to a specific FTD gene.

**TABLE 1:** Summary of genotype–phenotype correlations for gene defects most commonly associated with familial FTD.

**Gene**	**Suggestive features**
MAPT	- bvFTD, FTD with parkinsonism, (PPA)
	- No MND
	- AAO 48–55 years
	- Disease duration 9 years
GRN	- bvFTD (with apathy, social withdrawal), nfv-PPA
	- Presence of hallucinations, apraxia and amnestic syndrome
	- Presence of extrapyramidal symptoms; no MND
	- Asymmetric atrophy and fast rate of whole brain atrophy on MRI
	- Low serum progranulin
	- AAO 53–65 years
	- Disease duration 5–8 years
C9orf72	- bvFTD (nfv-PPA)
	- Presence of MND
	- Presence of psychiatric symptoms, bizarre behaviors, delusions, OCD-like behaviors
	- AAO 50–64 years
	- Disease duration 2.5–14 years, dependent on ALS comorbidity
	- Possible disease anticipation
TBK1	- bvFTD (with disinhibition, socially inappropriate behavior), (nfv-PPA, lv-PPA)
	- Presence of MND
	- Presence of extrapyramidal symptoms, early memory impairment, psychiatric symptoms
	- Asymmetric atrophy on MRI AAO 60–64 years
	- Disease duration 4–8 years
CHMP2B	- bvFTD with early personality change, disinhibition
	- Presence of parkinsonism, dystonia, pyramidal signs and myoclonus; no MND
	- AAO 58 years
VCP	- bvFTD (with apathy, emotional blunting, loss of initiative), SD
	- Presence of IBM, PDB
	- Presence of early psychosis, schizophrenia
	- AAO 48–65 years
	- Disease duration 6.5 years

### MAPT

Mutations in *MAPT*, located on chromosome 17, result in aberrant ratios of two of the six physiological isoforms of the tau protein. This disturbance in the equilibrium results in a disordered function of the cytoskeleton, affecting neuronal plasticity and axonal transport across the microtubules. It also leads to pathological tau aggregates, causing FTLD-tau. This association is not absolute, as *MAPT* mutations have been reported in the molecular pathogenetic pathways of PSP, CBD and, rarely, argyrophilic grain disease. The neuropathological correlations in these neurodegenerative brain disorders are distinct entities, with specific characteristics of the inclusion bodies as well as different localizations and distributions (Sieben et al., 2012).

FTLD-tau usually presents as bvFTD with mostly disinhibition, repetitive and stereotyped behaviors, or as FTD with parkinsonism, although PPA variants have been reported (Goldman et al., 2011; Seelaar et al., 2011; Sieben et al., 2012; Snowden et al., 2015; Che et al., 2018). Clinical heterogeneity is considerable, between and within families. Patients are frequently misdiagnosed with AD (Rademakers et al., 2004; Goldman et al., 2011). MND symptoms are uncommon. Symptoms develop at a particularly young age [average age 48–55 years (Seelaar et al., 2008, 2011; Goldman et al., 2011; Quaid, 2011; Sieben et al., 2012), and range 25–65 years (Goldman et al., 2011)]. Disease duration is 9 years on average (range: 5–20 years) (Seelaar et al., 2011; Sieben et al., 2012).

### GRN

*GRN*, located on chromosome 17, neighboring *MAPT*, encodes for progranulin which is a multifunctional growth factor involved in cell proliferation, wound healing and inflammation regulation (Che et al., 2018). Loss-of-function (LOF) mutations in *GRN* lead to autosomal dominant FTD (Baker et al., 2006; Cruts et al., 2006; Sieben et al., 2012; Che et al., 2018), as they reduce progranulin levels by 50% resulting in *GRN* haploinsufficiency (Van Mossevelde et al., 2018). *GRN* carriers generally present at autopsy with FTLD-TDP type A (Beck et al., 2008; Sieben et al., 2012).

The clinical phenotype of *GRN* carriers is highly variable. Most commonly, patients present with bvFTD, frequently showing apathy and social withdrawal (Rademakers et al., 2007; Le Ber et al., 2008; Seelaar et al., 2011; Sieben et al., 2012; Irwin et al., 2015; Che et al., 2018; Van Mossevelde et al., 2018). Memory impairment is an early symptom (Rademakers et al., 2007; Goldman et al., 2011). Several *GRN* carriers present with nfv-PPA and, less often, with a syndrome resembling lv-PPA (Rademakers et al., 2007; Beck et al., 2008; Sieben et al., 2012; Irwin et al., 2015; Snowden et al., 2015; Van Mossevelde et al., 2018). One study reported a remarkably high proportion of PPA presentations, outnumbering bvFTD (Van Mossevelde et al., 2016). Parietal lobe dysfunction and atrophy are characteristic features of *GRN* carriers, as well as marked asymmetry and a fast rate of whole brain atrophy on MRI (Beck et al., 2008; Goldman et al., 2011; Rohrer and Warren, 2011; Whitwell et al., 2015). Extrapyramidal symptoms are common, while signs of MND are rare. Diagnosis of AD and parkinsonian disorders associated with *GRN* have been reported (Rademakers et al., 2007; Le Ber et al., 2008; Goldman et al., 2011; Seelaar et al., 2011; Sieben et al., 2012; Irwin et al., 2015; Che et al., 2018; Van Mossevelde et al., 2018). Hallucinations, apraxia and amnestic syndrome may be more specifically associated with *GRN* mutations (Le Ber et al., 2008; Seelaar et al., 2011).

The average onset age for *GRN* mutation-caused FTD is 53–65 years although highly variable (range: 35–89) (Rademakers et al., 2007; Beck et al., 2008; Seelaar et al., 2008, 2011; Goldman et al., 2011; Sieben et al., 2012; Van Mossevelde et al., 2018; Wauters et al., 2018). The disease duration is shorter in *GRN* carriers than in *MAPT* carriers (average: 5–8 years) (Beck et al., 2008; Seelaar et al., 2011; Sieben et al., 2012; Van Mossevelde et al., 2018).

### C9orf72

The repeat expansion mutation in *C9orf72*, located on chromosome 9, is a major causal factor in the pathogenesis of both FTLD and ALS, forming a disease spectrum (Gijselinck et al., 2016). The hexanucleotide repeat of G_4_C_2_ is expanded in patients and is generally considered to be pathological when the expansion contains ≥ 2–24 repeat units (Renton et al., 2011; Gijselinck et al., 2012, 2018; Van Mossevelde et al., 2018). The exact mechanism of disease is unclear so far. Pathology may be due to haploinsufficiency or to gain-of-function, with toxic accumulation of the protein translated from the G_4_C_2_ repeat expansion as well as toxicity from the sense and antisense RNA foci transcribed from it (Gijselinck et al., 2012, 2016, 2018; Sieben et al., 2012; Van Mossevelde et al., 2018).

At the neuropathology level, *C9orf72* expansion carriers mostly have FTLD-TDP type A or B. Rarely, FTLD-UPS and FTLD-TDP type C were found (Sieben et al., 2012; Van Mossevelde et al., 2018). Clinically, *C9orf72* expansion carriers display a wide array of symptoms. Clinical heterogeneity of patient carriers is seen between and within families (Mahoney et al., 2012; Sieben et al., 2012; Van Mossevelde et al., 2018). FTD and ALS phenotypes frequently exist alone, while a combination of FTD and ALS symptoms has been reported in 17–30% of the *C9orf72* carriers (Sieben et al., 2012; Van Mossevelde et al., 2018). When FTD is present, it is mostly bvFTD (>65% of cases) with an early manifestation of executive dysfunction and some memory dysfunction, although PPA (most often nfv-PPA) has also been described (up to 30%) (Gijselinck et al., 2012; Mahoney et al., 2012; Sieben et al., 2012; Van Mossevelde et al., 2018). Symptoms of abnormal behavior are also frequently observed, like delusions, repetitive and typically complex behaviors that mimic obsessive-compulsive disorder (OCD) and irrational, bizarre behaviors. There is an absence of the increased sweet food preference as typically seen in bvFTD (Snowden et al., 2015; Van Mossevelde et al., 2018). There is an especially high occurrence of psychiatric symptoms (Mahoney et al., 2012; Devenney et al., 2014; Irwin et al., 2015; Van Mossevelde et al., 2018) and associated parkinsonism is common (Devenney et al., 2014; Van Mossevelde et al., 2018). The *C9orf72* expansion has also been identified in patients clinically diagnosed with AD, PD or Huntington disease phenocopy and several other disorders (Van Mossevelde et al., 2018). This may partly be due to lack of typical neuroimaging features, which is not uncommon in carriers of the *C9orf72* expansion (Devenney et al., 2014).

The mean onset age of symptoms for FTD caused by *C9orf72* expansion ranges between 50 and 64 years, but may be anywhere between 27 and 83 years of age (Mahoney et al., 2012; Van Mossevelde et al., 2016, 2018). Disease anticipation with decreasing onset ages in younger generations through expansion of the repeat size has been reported (Sieben et al., 2012; Gijselinck et al., 2016; Van Mossevelde et al., 2017b, 2018). A significantly later onset age has been recorded in patients with a short (<80 units; mean 62 years) compared to a long (>80 units; mean 53 years) repeat size (Gijselinck et al., 2016). When analyzing parent-offspring pairs, an earlier onset (16 to 25 years) was reported in the younger generation. Evidence for intergenerational repeat amplification has also been found, with an increase in expansion size of about 1000 units between a parent to their offspring and an intergenerational increase in methylation level of the 5′ flanking CpG island (Gijselinck et al., 2016). One study analyzing onset ages in 36 families of *C9orf72* repeat expansion carriers showed significantly earlier mean onset ages across successive generations (Van Mossevelde et al., 2017b). Measuring of the exact repeat size has proved difficult because of its 100% GC content, its large size, somatic instability and the repetitive nature of its flanking sequences (Gijselinck et al., 2016; Van Mossevelde et al., 2017a). This generates technical difficulties in measuring repeat sizes, requiring large quantities of high molecular weight genomic DNA (Van Mossevelde et al., 2017a). Recent novel technologies have enabled an increased resolution of the *C9orf72* expansion including the use of long-read sequencing (Ebbert et al., 2018).

The disease duration in *C9orf72* repeat expansion carriers is strongly dependent on ALS comorbidity (Van Mossevelde et al., 2018). In pure FTD, progression is slow (Devenney et al., 2014), average disease duration of 14 years was reported, which is much higher than the 2.5–3.6 years in pure ALS, resulting in a wide range of possible disease duration (1.7–22 years) (Mahoney et al., 2012; Sieben et al., 2012; Van Mossevelde et al., 2018).

### TBK1

*TBK1*, localized on chromosome 12, encodes the TBK1 protein, a serine-threonine kinase involved in autophagy, neuroinflammation, and phosporylation of a wide range of substrates. LOF mutations in *TBK1* lead to 50% reduction of TBK1, which is associated with clinical ALS and FTD, and inherited in families in an autosomal dominant pattern (Freischmidt et al., 2015; Gijselinck et al., 2015; Van Mossevelde et al., 2016, 2018; van der Zee et al., 2017). The associated underlying pathology is FTD-TDP (Freischmidt et al., 2015; Van Mossevelde et al., 2016).

Over 50% of *TBK1* carriers have a clinical presentation of MND. About 25% present with pure FTD, mostly bvFTD (>60%) but also nfv-PPA and lv-PPA (Gijselinck et al., 2015; Van Mossevelde et al., 2016, 2018). Disinhibition and socially inappropriate behavior are more frequent than apathy (Van Mossevelde et al., 2016). Extrapyramidal signs are common (Gijselinck et al., 2015; Van Mossevelde et al., 2016, 2018), as are early impairment of memory and psychiatric symptoms (Van Mossevelde et al., 2016). Structural MRI often shows marked asymmetry in atrophy (Van Mossevelde et al., 2016).

A mean age at onset of 60–64 years (range: 35–78 years) has been reported in TBK1 carriers and disease duration ranged from 1 to 16 years, with an average of 4–8 years (Freischmidt et al., 2015; Gijselinck et al., 2015; Van Mossevelde et al., 2016, 2018; van der Zee et al., 2017).

### Less Common FTD Genes

#### CHMP2B

*CHMP2B*, located at chromosome 3p11.2, encodes a component of the heteromeric ESCRT-III complex with functions in the endosomal–lysosomal and the autophagic protein degradation pathway (van der Zee et al., 2008; Urwin et al., 2010). Rare mutations were identified that resulted in a premature stop codon and C-truncating of the protein (van der Zee et al., 2008; Isaacs et al., 2011). Neuropathologically, *CHMP2B* carriers are associated with FTLD-UPS proteinopathy (van der Zee et al., 2008; Urwin et al., 2010; Goldman et al., 2011; Sieben et al., 2012).

Clinically, *CHMP2B* carriers present commonly with bvFTD with early personality changes, frequently represented by less concern for others, an unkempt appearance, disinhibition, inappropriate emotional responses and restlessness which later can be accompanied by aggression. Apathy, hyperorality and motor symptoms such as parkinsonism, dystonia, pyramidal signs, and myoclonus occur later. MND is typically not present, although some cases have been reported (Goldman et al., 2011; Isaacs et al., 2011; Seelaar et al., 2011; Sieben et al., 2012). PPA syndromes have been described as well (Isaacs et al., 2011; Sieben et al., 2012). The average onset age is 58 years, ranging between 46 and 65 years (Sieben et al., 2012).

#### VCP

*VCP*, located on chromosome 9 at 9p13.3, is associated with impaired functioning of an ATPase with a wide range of cellular functions. This impairment is due to missense mutations, of which > 30 have been identified so far (van der Zee et al., 2009; Cruts et al., 2012; Meyer and Weihl, 2014). Pathogenesis may occur because of a disturbance in the ubiquitin–proteasome mediated protein degradation, autophagy, or both (van der Zee et al., 2009; Sieben et al., 2012). The associated neuropathological correlation is FTLD-TDP type D (Sieben et al., 2012; Irwin et al., 2015).

*VCP* carriers present with a specific clinical syndrome, combining FTD (present in 30% of cases) with inclusion body myopathy (IBM) (present in 90% of cases) and Paget’s disease of the bone (PDB) (present in 50% of cases) in inclusion body myopathy with early onset Paget’s disease and frontotemporal dementia (IBMPFD). Presentations may include any or all of these clinical entities, creating a disease spectrum. FTD symptoms usually fall within the category of bvFTD (with apathy, emotional blunting and loss of initiative and spontaneity) and sv-PPA (Sieben et al., 2012; Van Mossevelde et al., 2018). Psychotic signs and schizophrenia are common early symptoms. Parkinsonism is not uncommon (Van Mossevelde et al., 2018). Other neurological diagnoses in *VCP* mutation carriers include PD, AD and, rarely, peripheral sensorimotor neuropathy, Charcot–Marie–Tooth disease type 2 and hereditary spastic paraplegia (Van Mossevelde et al., 2018).

Age at onset is 48–65 years (range: 39–73 years), with a disease duration of 6.5 years (Goldman et al., 2011; Sieben et al., 2012; Van Mossevelde et al., 2018). Onset age of FTD is considerably later than that of IBM and PDB (Van Mossevelde et al., 2018).

## Future Biomarkers for Improved Diagnosis of FTD

### Imaging Biomarkers in a Research Setting

#### Diffusion Tensor Imaging

Diffusion tensor imaging (DTI) is an MRI imaging technique visualizing the diffusion of water molecules throughout the brain. It is used as a technique for white matter tractography (Mahoney et al., 2014).

Studies have shown that white matter damage is an early marker for disease in FTD, and DTI may be used as a tool to screen for such abnormalities at the presymptomatic stage (Dopper et al., 2014; Mahoney et al., 2014; Jiskoot et al., 2019). Reduced integrity of the uncinate fasciculus and anterior corpus callosum is typical for FTD, and the degree of damage is correlated to age and disease severity (Dopper et al., 2014; Jiskoot et al., 2019). Even here, specific patterns can be recognized for different clinical subtypes, and for carriers of different causal mutations. These abnormalities are consistent with characteristic brain atrophy distributions (Dopper et al., 2014; Lam et al., 2014; Agosta et al., 2015; Jiskoot et al., 2019). The increase in white matter damage over time has been reported to be greater than that of gray matter atrophy, although only at the symptomatic stage, indicating the possible use of this technique as a marker for disease progression (Lam et al., 2014; Jiskoot et al., 2019).

Studies comparing FTD cohorts with AD patients and with normal controls found significantly more white matter pathology mostly in bilateral uncinate fasciculus, cingulum bundle, and corpus callosum in FTD compared to both other groups (Mahoney et al., 2014; Bang et al., 2015). More studies are needed to consolidate these findings and define the diagnostic accuracy for FTD of DTI, as well as its power to distinguish FTD from other types of neurodegenerative brain diseases.

#### Resting-State fMRI

In resting-state functional MRI (fMRI), regional connectivity is measured through fluctuations in blood-oxygen-level dependent (BOLD) signal. FTD patients, most often those with bvFTD, have decreased functional connectivity mostly in the SN, necessary for emotional processing, behavior and interpersonal experiences (Greicius, 2008; Zhou et al., 2010; Dopper et al., 2014). A distinction can be made between FTD and AD, as in the latter a different pattern of loss of functional connectivity is seen involving the default mode network (Greicius, 2008; Zhou et al., 2010). The changes on fMRI are thought to be measurable at the presymptomatic stage. However, more research is needed to allow for a large scale application of this technique (Dopper et al., 2014).

#### Arterial Spin Labeling

Arterial spin labeling (ASL) is an MRI technique which, like SPECT, measures cerebral blood flow. It does so by magnetically labeling water molecules and has the advantages of being a method of functional brain imaging that is non-invasive and cost-effective, as no tracer molecule is necessary and it can easily be added to a routine structural MRI scan (Grade et al., 2015).

One of its clinical applications is the identification of regional hypoperfusion in neurodegenerative disease (Du et al., 2006; Bron et al., 2014; Verfaillie et al., 2015; Steketee et al., 2016). Sensitivity and specificity to differentiate FTD from healthy controls have been reported as 78–79 and 76–92%, respectively. When distinguishing FTD from AD, these results were 69–83 and 68–93% (Steketee et al., 2016; Tosun et al., 2016). The accuracy in differentiating FTD from AD when ASL results are combined with structural MRI has been reported as 87% (Du et al., 2006). However, its added value compared to structural MRI alone might be limited (Bron et al., 2014). In direct comparison to FDG PET, ASL came out as comparable (Verfaillie et al., 2015; Tosun et al., 2016).

#### Tau PET Imaging

In recent years, amyloid PET scan measuring amyloid-β burden with tracers such as [C-11] Pittsburgh Compound B (PIB), flutemetamol, florbetapir (AV-45), florbetaben (AV-1), and AZD4694 has proven its value in quantifying underlying AD neuropathology. It is a well-established technique to confirm or rule out AD with great diagnostic accuracy. Abnormalities are present years before onset of symptoms, and this biomarker can be used as a measure for staging and monitoring of disease progression and distribution (Klunk et al., 2004; Dubois et al., 2007, 2014; Rowe et al., 2007; Jack et al., 2010).

No such imaging technique exists, yet, to confirm or rule out FTD. An extra hurdle here is the heterogeneity in underlying protein inclusions, as we mentioned earlier. For patients with an underlying FTLD-tau pathology, however, novel PET imaging techniques are being developed. The nature of the pathophysiology heightens the challenge in developing a useful ligand, as tau is a protein existing in six isoforms that undergo complex post-translational modifications. Moreover, the pathological tau aggregates [neurofibrillary tangles (NFTs)] are located intracellularly (Villemagne et al., 2017). Ligands such as [18F]THK523, [18F]THK5117, [18F]THK5105 and [18F]THK5351, [18F]AV1451(T807) and [11C]PBB3 have been proven to show the distribution of tau pathology *in vivo*. However, NFT are common in the pathophysiology of many other neurodegenerative brain diseases, such as AD, PSP, CBD, and chronic traumatic encephalopathy (Dani et al., 2016). Tau PET imaging also does not have the advantage of showing abnormalities at a presymptomatic stage, as there is a temporal relationship between tau-PET and symptoms. There is, however, a correlation between quantitative NFT burden and cognitive decline. Hence, through more research, tau PET imaging might be of use as a valuable marker for disease progression, staging and possibly therapeutic response (Dani et al., 2016; Villemagne et al., 2017).

#### Electroencephalography

Electroencephalography is a non-invasive, simple, widely accessible technique that can be used to measure the physiological functionality of the brain, and disruption thereof in neurodegenerative brain disease (Adamis et al., 2005; Goossens et al., 2017).

Through quantitative analysis of EEG aberrations as opposed to more commonly carried out visual assessment, a more robust diagnostic value of this biomarker can be achieved. Accuracy of differentiating FTD from AD in patients with moderate to severe dementia has been reported as 79–100%, with a significantly lower frequency of the dominant frequency peaks in AD than in FTD, amongst other findings (Garn et al., 2017; Goossens et al., 2017). Distinguishing FTD from healthy controls has proven more difficult, as EEG in FTD patients is usually relatively normal, especially in the early disease stages (Goossens et al., 2017).

#### Transcranial Magnetic Stimulation

Transcranial magnetic stimulation (TMS) is a painless, non-invasive procedure that assesses the cortical circuits and their function. An electromagnetic coil is placed on the scalp, where it generates a magnetic field. This induces a measurable electrical current in the brain, depolarizing the cells (Guerra et al., 2011).

This technique has shown some value in the differential diagnosis of FTD, although results differ, cohorts are small and methodologies are difficult to compare (Pierantozzi et al., 2004; Alberici et al., 2008; Issac et al., 2013; Benussi et al., 2016, 2017). The study with the largest scale so far is an Italian trial, where TMS differentiated FTD (*n* = 64) from AD (*n* = 79) with a sensitivity of 92% and a specificity of 89%, and FTD from healthy controls (*n* = 32) with a sensitivity of 90% and a specificity of 78% (Benussi et al., 2017). However, their setup was only partially blinded, as the operator knew whether the subject was a healthy control or a patient, but not what clinical diagnosis had been given. The major differences in both diseases were an impairment of the short interval intracortical inhibition/intracortical facilitation (SICI-ICF) in FTD and an impairment of the short-latency afferent inhibition in AD (Cantone et al., 2014; Benussi et al., 2017). The same group also published findings of a distinct difference between healthy controls and presymptomatic *GRN* mutation carriers (Benussi et al., 2016).

These results point to TMS as a possible early, non-invasive tool for diagnosis of FTD. More research is needed to consolidate this evidence.

### Neurochemical Biomarkers in a Research Setting

#### Neurofilament Light Chain

Neurofilament light chain (NfL) is a new candidate CSF and serum biomarker for the diagnosis of FTD. Neurofilaments are structural axonal proteins, and their presence in CSF is a marker for neurodegeneration (Goossens et al., 2018).

Several studies have reported that CSF NfL levels are significantly increased in both FTD (se 78–84%, sp 82–100%) (Meeter et al., 2016, 2018; Abu-Rumeileh et al., 2018) and AD in comparison with controls (Goossens et al., 2018). However, NfL levels have been shown to be significantly higher in FTD than in AD, adding to the diagnostic accuracy achieved with a classic AD CSF biomarker panel alone (Abu-Rumeileh et al., 2018; Goossens et al., 2018). Pathologically confirmed FTLD-TDP patients have been reported as having higher NfL levels than those with FTD-tau (se 80%, sp 81%) (Rohrer et al., 2016; Abu-Rumeileh et al., 2018), although other studies could not confirm these results (Goossens et al., 2018; Meeter et al., 2018).

Evidence for a good correlation between CSF and serum NfL levels exists, with a similar elevation in patients compared to healthy controls (Meeter et al., 2016; Rohrer et al., 2016) and presymptomatic causal mutation carriers (Meeter et al., 2016). NfL may also serve as a biomarker for disease severity and even rate of disease progression, and for conversion to the symptomatic stage in carriers of causal mutations (Meeter et al., 2016; Rohrer et al., 2016).

#### TDP-43

As mentioned earlier, one of the molecular pathways leading to FTLD neuropathology is the intraneuronal aggregation of TDP-43, accounting for approximately half of all FTLD cases (Goossens et al., 2015). The pathological protein has been proposed as a potential CSF biomarker specific to FTLD (Goossens et al., 2015). Some difficulties have arisen, however. Because of low absolute levels of TDP-43 in biofluids, a very sensitive immunoassay is required, preferably specific for pathological TDP-43. Many different assays have already been developed, but their sensitivity and specificity are not yet well-established (Goossens et al., 2015). Another obstacle is the occurrence of TDP-43 co-pathology in other neurodegenerative brain diseases, which may be present in 20–56% of patients with AD and other tauopathies, undermining the specificity of the biomarker (Arai et al., 2009; Goossens et al., 2015).

#### Progranulin

As we mentioned earlier, LOF mutations in *GRN* result in progranulin haploinsufficiency. Consequent decreased progranulin levels are detectable in plasma and CSF of carriers of such a causal mutation in *GRN*, both in patients with clinical FTD as in presymptomatic carriers, with a sensitivity and specificity of 100% (Ghidoni et al., 2008; Finch et al., 2009; Sleegers et al., 2009; Goossens et al., 2018).

Thus, dosage of plasma progranulin is a potential cheap and non-invasive tool for screening for carriers of genetic *GRN* defects. It is the only FTD-specific biomarker so far. However, given the small number of all FTD patients (both familial and sporadic) that carry a *GRN* mutation, its use may not be indicated for every patient in the initial diagnostic workup. The biomarker may be more useful in patients with a strong family history of FTD, although further genetic testing may then be required afterward, or to identify presymptomatic carriers amongst asymptomatic family members identified to have a causal *GRN* mutation. One must also take into account the region of origin of the patient, as *GRN* mutations represent a much larger share of all autosomal dominant FTD in certain regions (e.g., Belgium, Italy) than in others due to a founder effect (van der Zee et al., 2006; Rademakers et al., 2007; Benussi et al., 2013). This factor may provide an additional indication for measuring plasma progranulin.

## Conclusion: Recommendations for Daily Clinical Practice

**FIGURE 3:**
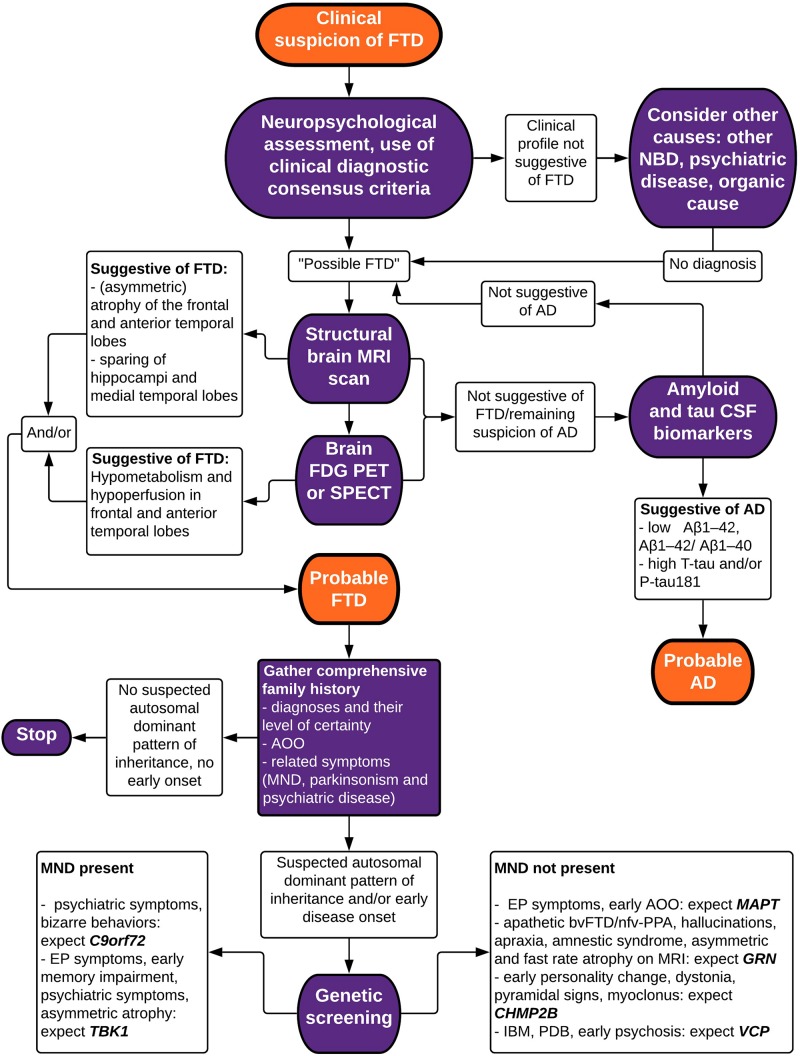
Flowchart depicting a stepwise approach to the diagnosis of FTD. bv, behavioral variant; FTD, frontotemporal dementia; nfv-PPA, non-fluent variant primary progressive aphasia; AD, Alzheimer’s disease; MND, motor neuron disease; NBD, neurodegenerative brain disease; PSP, progressive supranuclear palsy; IBM, inclusion body myopathy; PDB, Paget’s disease of the bone; MRI, magnetic resonance imaging; FDG PET, fluorodeoxyglucose positron emission tomography; SPECT, single-photon emission computed tomography; CSF, cerebrospinal fluid; EP, extrapyramidal; AAO, age at onset.

Making a correct and well-founded diagnosis of FTD is not an easy task, due to clinical heterogeneity and overlap with other neurodegenerative brain diseases. Atypical presentations are common, which is exemplified by our clinical vignette. A wide range of techniques exist to aid in the differential diagnosis, identifying characteristics of neuropsychology and biomarkers of neuroimaging, neurochemistry, and genetics. In this review paper, we have summarized current evidence for the diagnostic value of these techniques in differentiating FTD from other neurodegenerative diseases (mainly AD), and in differentiating between FTD subtypes. Taking this knowledge into account, we propose a stepwise approach in the diagnostic evaluation of a patient suspected to suffer from FTD (Figure 3).

Firstly, obtaining the patient history and a standardized neuropsychological testing are necessary to establish the presence of a typical neuropsychological profile suggestive of FTD. Clinical consensus criteria for bvFTD as well as for PPA variants of FTD have a reasonably good accuracy in differentiating FTD from other diagnoses, although misdiagnosis (especially misidentifying FTD as AD and vice versa) is common. They aid in guiding the clinician’s view by standardizing and quantifying the presenting symptoms and play a major role in establishing the correct diagnosis.

A second step indicated in any patient with a presentation of possible neurodegenerative brain disease is the acquisition of neuroimaging biomarkers. Structural brain MRI is most commonly used and typically shows a more pronounced loss of volume in the frontal lobes and the anterior temporal lobes. Asymmetry and sparing of the hippocampi and medial temporal lobes are accurate markers to differentiate FTD from AD. Evidence also shows that specific patterns of brain atrophy are associated with different clinical, neuropathological, and genetic subtypes of FTD. An important advantage of brain MRI is the good resolution in visualizing the brain tissue, with a possibility to screen for other organic causes of symptoms, such as brain tumors and hydrocephalus, and to quantify vascular lesions, making this a great choice as a first screening diagnostic technique. In recent years, novel techniques such as automatic volumetry, VBM, TBM, manifold learning, ROI-based grading, automated measurement of vascular burden and of cortical thickness have been developed to add to the diagnostic accuracy of structural MRI.

Functional neuroimaging through brain FDG PET or perfusion SPECT scans can also be considered in the initial diagnostic workup, as studies have shown high sensitivity and specificity to diagnose FTD. Hypometabolism and relative hypoperfusion are typically seen in the frontal and anterior temporal lobes, usually correlating with atrophy on MRI but often preceding it. Some studies have shown a greater accuracy of FDG PET when compared to SPECT, but the evidence is not very robust. For the clinician, the local availability of the techniques as well as cost-effectiveness (with SPECT being more affordable) also need to be taken into account.

When these imaging biomarkers are not sufficient to establish a clear diagnosis, and certainly in case of differential diagnostic doubt with (atypical presentation of) AD, we suggest a further exploration through the analysis of CSF biomarkers Aβ_1__–__42_, Aβ_1__–__42_/Aβ_1__–__40_ ratio, T-tau and P-tau_181_ that can indicate the presence of AD with good accuracy. FTD cannot be diagnosed based on CSF biomarkers, but non-specific, intermediate decreased Aβ_1__–__42_ and increased T-tau levels may or may not be present. The use of P-tau_181_ and the Aβ_1__–__42_/Aβ_1__–__40_ ratio significantly increases the accuracy of correctly identifying FTD vs. AD.

Lastly, we have elaborated on the genetic basis of FTD. To ascertain the possible presence of a causal mutation for FTD, a comprehensive family history complete with diagnoses, their level of certainty, age at onset and possible other related symptoms such as MND, parkinsonism and psychiatric disease should be inquired. When an autosomal dominant pattern of inheritance is suspected upon pedigree analysis or when the patient presents with an early onset age, screening for causal mutations may be indicated in the affected patient. We have summarized genotype–phenotype correlations for the most commonly involved genes (*C9orf72*, *MAPT*, *GRN, TBK1*) and some less frequent ones (*CHMP2B*, *VCP*), including average age at onset and disease duration (Table 1). We also mentioned the neuropathological correlation expected with each gene defect, which may be of particular interest when new disease-modifying therapies become available.

More research into novel biomarkers for the diagnosis of FTD and other neurodegenerative brain diseases is needed. Above, we have summarized current evidence on some novel imaging biomarkers (DTI, resting state fMRI, ASL, tau pet imaging, EEG, TMS) as well as neurochemical biomarkers (NfL, TDP-43, progranulin) (Table 2). These promising techniques are currently still only used in an experimental setting, or are not yet routinely implemented for screening in patients with suspected FTD.

**TABLE 2:** Summary of potential future biomarkers to diagnose FTD.

**Imaging biomarkers in a research setting**	
DTI	- MRI technique, visualizes white matter damage
	- Damage in bilateral uncinate fasciculus, cingulum bundle, and corpus callosum are considered typical for FTD
	- Possibly also useful in presymptomatic stage
Resting state fMRI	- Measures regional connectivity through BOLD signal
	- Decreased connectivity in SN typical for FTD
	- Possibly also useful in presymptomatic stage
ASL	- Functional MRI technique, measures perfusion
	- Non-invasive, cost-effective (compared to FDG PET and perfusion SPECT)
	- Good diagnostic accuracy
Tau PET imaging	- Quantifies NFT burden
	- Search for optimal ligand still ongoing
	- Abnormalities expected to be present in all tauopathies, not specific to FTLD
	- Possibly also useful for staging and as marker for disease progression
EEG	- Not invasive, widely accessible
	- Good accuracy for differential diagnosis FTD-AD in moderate to severe dementia, more evidence required in early stage
TMS	- Measures cortical circuitry through external electromagnetic coil
	- Impairment of SICI-ICF typical for FTD
	- Possibly also useful in an early stage
**Neurochemical biomarkers in a research setting**	
NfL	- Marker suggestive of neurodegeneration
	- Measurable in both CSF and serum / plasma
	- Good diagnostic accuracy in research but clinical validation needed
	- Possibly also useful for staging and as marker for disease progression
TDP-43	- Marker of TDP-43 neuropathology
	- Specific to FTLD-TDP, although TDP co-pathology occurs in other neurodegenerative brain diseases
	- Search for optimal antibody still ongoing
Progranulin	- Marker for *GRN* LOF mutation
	- Measurable in both CSF and plasma
	- 100% sensitive and specific to *GRN* mutation carriers
	- Indicated for screening in (pre)symptomatic possible carriers of *GRN* defects

## Author Contributions

SE, HG, and CVB conceived of the presented idea. HG wrote the manuscript with support of and critical reading by SE and CVB.

## Conflict of Interest Statement

The authors declare that the research was conducted in the absence of any commercial or financial relationships that could be construed as a potential conflict of interest. The reviewer LB declared a past co-authorship with two of the authors CVB and SE to the handling Editor.
